# Antioxidant and Anti-Inflammatory Mechanisms of Lipophilic Fractions from *Polyscias fruticosa* Leaves Based on Network Pharmacology, In Silico, and In Vitro Approaches

**DOI:** 10.3390/foods12193643

**Published:** 2023-10-01

**Authors:** Razanamanana H. G. Rarison, Van-Long Truong, Byoung-Hoon Yoon, Ji-Won Park, Woo-Sik Jeong

**Affiliations:** 1 School of Food Science & Biotechnology, College of Agriculture and Life Sciences, Kyungpook National University, Daegu 41566, Republic of Korea; 2 Food and Bio-industry Research Institute, School of Food Science & Biotechnology, College of Agriculture and Life Sciences, Kyungpook National University, Daegu 41566, Republic of Koreatruonglongpro@gmail.com (V.-L.T.);

**Keywords:** *Polyscias fruticosa* leaf, network pharmacology, molecular docking, nutraceuticals, antioxidant, anti-inflammation

## Abstract

*Polyscias fruticosa* leaf (PFL) has been used in food and traditional medicine for the treatment of rheumatism, ischemia, and neuralgia. However, the lipophilic components of PFL and their biological properties remain unknown. This study, integrating network pharmacology analysis with in silico and in vitro approaches, aimed to elucidate the antioxidant and anti-inflammatory capacities of lipophilic extracts from PFL. A total of 71 lipophilic compounds were identified in PFL using gas chromatography–mass spectrometry. Network pharmacology and molecular docking analyses showed that key active compounds, mainly phytosterols and sesquiterpenes, were responsible for regulating core target genes, such as PTGS2, TLR4, NFE2L2, PRKCD, KEAP1, NFKB1, NR1l2, PTGS1, AR, and CYP3A4, which were mostly enriched in oxidative stress and inflammation-related pathways. Furthermore, lipophilic extracts from PFL offered powerful antioxidant capacities, as evident in our cell-free antioxidant assays. These extracts also provided a protection against oxidative stress by inducing the expression of catalase and heme oxygenase-1 in lipopolysaccharide (LPS)-treated RAW 264.7 cells. Additionally, lipophilic fractions from PFL showed anti-inflammatory potential in downregulating the level of pro-inflammatory factors in LPS-treated macrophages. Overall, these findings provide valuable insights into the antioxidant and anti-inflammatory properties of lipophilic extracts from PFL, which can be used as a fundamental basis for developing nutraceuticals and functional foods.

## 1. Introduction

Oxidative stress refers to disruption in the equilibrium between reactive oxygen species (ROS) and reactive nitrogen species (RNS) production, and their elimination by endogenous antioxidants due to the extended production of oxidants. As ROS/RNS are molecules each containing an unpaired electron, they can readily react with biological macromolecules, including lipids, proteins, and DNA [1]. Consequently, the overproduction of ROS/RNS can alter the normal functions of cells, cause tissue injury and, subsequently, promote the initiation and progression of multiple diseases, such as diabetes, cardiovascular diseases, aging, and cancer [2]. Therefore, cellular redox homeostasis helps in maintaining the normal physiological processes of living organisms and preventing the onset of chronic disorders.

Inflammation is a protective immune response against exogenous or endogenous harmful stimuli and oxidative stress to maintain homeostasis in an organism [3]. However, the exaggerated and long-lasting release of inflammatory cytokines and mediators, such as cyclooxygenase-2 (COX-2), inducible nitric oxide synthase (iNOS), interleukin (IL)-1β, IL-6, or tumor necrosis factor-alpha (TNF-α), leads to molecular dysfunction, extensive oxidative stress, and the onset and progression of chronic disease [4]. Furthermore, accumulating evidence has demonstrated an intercommunication between oxidative stress and inflammation, in which oxidative stress triggers inflammation by activating a redox transcription factor, namely NF-κB, while the inflammatory response enhances the accumulation of radicals/oxidants [5,6]. Thus, there is growing interest in the search for natural compounds that could mitigate the effects of the inextricable relationship between inflammation and oxidative stress.

*Polyscias fruticosa*, belonging to the family *Araliaceae*, is a perennial shrub native to tropical regions and commonly cultivated in Vietnam and India. *P. fruticosa* leaves have been used in food as salad and in folk medicine for the treatment of rheumatoid arthritis, neuralgia, dysentery, and ischemia [7]. To date, emerging studies have revealed the biological properties of *P. fruticosa* leaf (PFL), particularly ethanolic or hydrophilic extracts; these properties include antipyretic and analgesic activity, the prevention of neurogenerative diseases, the mitigation of bone loss, and the alleviation of respiratory inflammation [7,8,9,10]. In addition, these health-promoting effects have been attributed to *P. fruticosa* active compounds, including saponins, polyphenols, flavonoids, tannins, and vitamins [7,9,11]. Although the volatile compounds in PFL have been previously profiled [12], their biological capacities remain unknown. Moreover, lipophilic fractions may provide distinct health benefits compared to hydrophilic fractions. They are rich fat-soluble antioxidants that can combat oxidative stress and inflammation, contributing to overall wellbeing. Additionally, lipophilic compounds offer a unique ability to effectively penetrate lipid-rich cellular membranes, which facilitates their interaction with intracellular targets. Therefore, the identification of lipophilic compounds in PFL and the elucidation of their functional properties are worthwhile aims.

Network pharmacology is a powerful and cost-effective bioinformatic tool that has emerged for deciphering the biological mechanisms of multiple compounds and their interactions with the biological systems of interest [13]. Overcoming the traditional “one drug, one target, one disease” concept, this approach allows for the holistic exploration of complex interactions between bioactive compounds and biological systems, facilitating the identification of potential targets and shedding light on the mechanisms underlying their therapeutic activity [14]. Furthermore, network pharmacology also enhances our understanding of the multifaceted effects of natural compounds, particularly plant extracts and herbal remedies. In addition, predictions from network-based methods are commonly validated through molecular docking, a valuable computational technique for predicting the binding affinities and interactions between small molecules, often those found in plant extracts, and specific protein targets [15]. Recently, these approaches have been used extensively to explore the underlying mechanisms of bioactive compounds, especially in complex herbal decoctions or crude extracts [16,17,18]. Hence, this study focused on investigating the functional properties of lipophilic extracts of PFL using an integrative approach of network pharmacology analysis with molecular docking and in vitro verifications. 

## 2. Materials and Methods

### 2.1. Materials

Lipopolysaccharide (LPS, *Escherichia coli* O127:B8), 2,2′-diphenyl-1-picrylhydrazyl radical (DPPH), 2,2′-azino-bis-3-ethylbenzothiazoline-6-sulfonic acid (ABTS), dimethyl sulfoxide (DMSO), Folin–Ciocalteu′s phenol reagent, 2,4,6-tris(2-pyridyl)-1,3,5-triazine (TPTZ), gallic acid, catechin, sodium nitroprusside, 3-(4,5-dimethylthiazol-2-yl)-2,5-diphenyltetrazolium bromide (MTT), 2′,7′-Dichlorodihydrofluorescein diacetate (DCFH-DA), and Trolox were purchased from Sigma-Aldrich (St. Louis, MO, USA). Trichloroacetic acid (TCA) and 12-molybdo(VI) phosphoric acid b-hydrate were obtained from Junsei Chemical Co. (Tokyo, Japan). Anti-COX-2, iNOS, and HRP-conjugated anti-rabbit secondary antibodies were purchased from Cell Signaling Technology (Danvers, MA, USA). Anti-IL-1β, CAT, CAT, SOD-2, and HRP-conjugated anti-mouse secondary antibodies were acquired from Santa Cruz Biotechnology (Dallas, TX, USA). All other reagents are of analytical grade.

### 2.2. Preparation and Chemical Composition Analysis of Lipophilic Fractions from P. fruticosa Leaves

Dried powder of *P. fruticosa* leaves (100 g) was extracted twice with 95% ethanol (1:10, *w*/*v*) at room temperature over 24 h. This solvent is widely used to effectively extract a broad range of plant constituents, including both lipophilic and hydrophilic compounds, due to its physicochemical properties and safety. After filtration, the filtrates were combined and concentrated using a rotary evaporator (Eyela, Tokyo, Japan) under vacuum conditions, yielding 13.46 ± 2.03 g of the extract. The ethanol crude extract (10 g) was resuspended in 200 mL of distilled water and sequentially fractionated with n-hexane three times (200 mL each) and dichloromethane three times (200 mL each). Each fraction was then evaporated using a rotary evaporator (Eyela) to obtain *P. fruticosa* leaf hexane (PFLH, 1.61 ± 0.31 g) and dichloromethane (PFLD, 1.11 ± 0.49 g) extracts.

Chemical compositions of PFLH and PFLD extracts were analyzed via gas chromatography (Agilent 7890A, Agilent Technologies, Santa Cruz, CA, USA) combined with mass spectrometry (Agilent 5975C) using an Agilent J&W DB-5ms fused silica capillary column (60 m × 0.25 mm × 0.25 μm). Helium was introduced as the carrier gas, with a constant flow of 1 mL/min. The oven temperature was programmed to be 50 °C for 3 min, then raised to 310 °C at the rate of 5 °C/min and, finally, maintained at 310 °C for 25 min. The injection temperature was 280 °C. Mass spectra were detected between 35 and 600 m/z, with an ionization energy of 70 eV and a full scan at the rate of 0.132 s/scan. The chemical compositions were identified via the comparison of their mass spectra with mass spectra in the Agilent MassHunter Library, which is composed of the National Institute of Standards and Technology (NIST) and Wiley Libraries.

### 2.3. Chemical-Based Antioxidant Activity Assays

The DPPH radical scavenging activity was assayed with a slight modification to the procedure reported previously [19]. The DPPH solution (200 μM dissolved in methanol) was added to the samples or Trolox solution up to 200 μL. Before the measurement of the absorbance at 517 nm, the mixtures were reacted in the dark for 30 min at room temperature. The result was calculated using the following equation:

Radical scavenging activity (%) = [(Abs_c_ − Abs_s_)/Abs_c_] × 100 (1)

where Abs_c_ is the absorbance of the control, and Abs_s_ is the absorbance of the samples.

The ABTS^•+^ radical scavenging activity was measured using a modified version of the method described previously [19]. To generate ABTS^•+^, the same volume of the 7.4 mM ABTS solution and 2.45 mM potassium persulfate was mixed and kept in the dark at room temperature for 14 h before use. Until an absorbance in the range of 0.70 ± 0.02 at 734 nm was reached, the ABTS^•+^ solution was diluted with ethanol. The sample and Trolox were added to the working ABTS^•+^ solution, and their absorbance was measured at 740 nm. Equation (1) was also used to estimate the ABTS^•+^ radical scavenging activity, expressed as IC_50_.

The superoxide anion radical (O_2_^•−^) scavenging activity was measured following pyrogallol autoxidation in an alkaline solution with minor modifications [20]. In brief, samples were mixed with 50 mM Tris-HCl buffer (pH 8.2) and maintained at 20 °C. After 20 min, 5 mM pyrogallol was added to the mixture. Trolox served as the positive control, and the reading taken at 325 nm was represented as % radical scavenging, as stated in Equation (1).

Griess reagent was used to assess the production of nitric oxide (NO) radicals from the reaction of sodium nitroprusside with oxygen [21]. In brief, 10 mM sodium nitroprusside solution was added to various concentrations of the sample or Trolox solution and maintained at 25 °C for 150 min. Then, for the assessment of the generated NO radicals, an equal amount of Griess reagent was added and left to react for 30 min. The absorbance was measured at 546 nm, and the percentage of inhibition was calculated as in Equation (1).

Hydrogen peroxide (H_2_O_2_) production was quantified based on the chemiluminescence enhancer method [22]. The sample or Trolox solution was mixed with 10 mM H_2_O_2_ and 250 μM luminol in 1 M sodium hydroxide. The generation of chemiluminescence was continuously measured for 10 min at 37 °C. The percentage of mitigation was calculated following Equation (1).

The production of hydroxyl radicals was quantified using a slightly modified version of the salicylic acid method [20]. In brief, different concentrations of the sample or Trolox solution were mixed with 20 mM salicylic acid and 1.5 mM iron chloride. Then, the Fenton reaction, resulting in hydroxyl radical generation, was induced through the addition of 60 mM hydrogen peroxide. The absorbance was determined at 510 nm after 1 h of incubation at 37 °C. Using Equation (1), the hydroxyl radical scavenging activity was expressed as a percentage.

All radical scavenging activities were expressed as a half-maximal inhibitory concentration (IC_50_).

A ferric reducing antioxidant power (FRAP) assay was conducted as previously reported, with a minor adjustment [20]. The FRAP reagent, composed of a mixture of 300 mM acetate buffer (pH 3.6), 10 mM TPTZ, and 20 mM FeCl_3_ (10:1:1, *v*/*v*/*v*) warmed at 37 °C for 10 min, was added to the sample or Trolox solution. After 30 min of incubation in the dark at room temperature, the absorbance was read at 590 nm, and the FRAP value was calculated as the Trolox equivalent (mg TE/g dried extract).

A potassium ferricyanide antioxidant reducing power (PFRAP) assay was conducted according to a previous study [23]. Accordingly, the sample or Trolox solution was mixed with 1% potassium ferricyanide and ethanol. Following incubation at 50 °C for 30 min, the mixtures were cooled down and 10% TCA was added, followed by centrifugation at 3000 rpm for 10 min. Then, ethanol and 0.1% FeCl_3_ were added to the supernatants before the reading was taken at 700 nm. The PFRAP value was quantified as the Trolox equivalent (mg TE/g dried extract).

The reduction of molybdenum (VI) to molybdenum (V) was carried out to determine the total antioxidant capacity (TAC) [23]. In brief, the working reagent [4 mM 12-Molybdo(VI) phosphoric acid n-hydrate, 0.6 M sulfuric acid, 28 mM sodium phosphate, 1:1:1, *v*/*v*/*v*] was added to the sample and Trolox solutions and left to react for 90 min at 95 °C. Before the reading was taken at 695 nm, the mixtures were cooled down. The result was expressed as the Trolox equivalent (mg TE/g dried extract).

### 2.4. Cell Culture

RAW 264.7 macrophages, purchased from ATCC (Rockville, MD, USA), were cultured in Dulbecco’s Modified Eagle Medium containing 10% fetal bovine serum, 100 U/mL penicillin, and 100 μg/mL streptomycin at 37 °C in a humidified incubator with 5% CO_2_.

### 2.5. Measurement of NO Production

RAW 264.7 cells were pretreated with different concentrations of PFLH or PFLD for 1 h before LPS (1 μg/mL) challenge for an additional 12 h. The extracellular NO production was quantified via mixing equal volumes of cell culture media and Griess reagent. The absorbance was measured at 546 nm, and the NO concentration was calculated using a NaNO_2_ standard curve.

### 2.6. Measurement of Reactive Oxygen Species (ROS) Formation

RAW 264.7 cells were pretreated with different concentrations of PFLH or PFLD for 1 h before LPS (1 μg/mL) challenge for an additional 6 h. The intracellular ROS generation was estimated using a fluorescent probe, DCFH-DA. In brief, after treatment, the cells were incubated with 20 μM DCFH-DA for 1 h in a 37 °C incubator. The excessive probes were then washed twice with phosphate-buffered saline, and the fluorescence intensities were measured at 485/20 nm of excitation and 525/20 nm of emission. 

### 2.7. Western Blot Analysis

Proteins from the treated cells were extracted using RIPA buffer (Cell Signaling), and the total protein concentration was quantified using a BCA protein assay kit (Thermo Scientific, Rockford, IL, USA). Equal amounts of protein samples were fractionated in sodium dodecyl sulfate polyacrylamide gels and then electro-blotted onto polyvinylidene fluoride membranes. After blocking with 5% non-fat milk for 2 h at room temperature, the membranes were hybridized with specific primary antibodies at 4 °C overnight and, subsequently, incubated with appropriate secondary antibodies for 3 h at 4 °C. The blots were visualized using a chemiluminescence reagent (ATTO, Tokyo, Japan).

### 2.8. Network Pharmacology Analysis

#### 2.8.1. Prediction of Potential Targets of Compounds in Lipophilic Extracts from *P. fruticosa* Leaves

The SMILES format for each compound in lipophilic extracts from *P. fruticosa* leaves was obtained from the PubChem database (https://pubchem.ncbi.nlm.nih.gov/ (accessed on 18 May 2023). The SMILES formats were inputted into SwissADME (http://www.swissadme.ch/ (accessed on 18 May 2023), and the compounds that complied with Lipinski’s rule were retained for subsequent analysis. The potential targets of compounds in lipophilic extracts from PFL were acquired from SwissTargetPrediction (https://swisstargetprediction.ch/ (accessed on 18 May 2023) and SuperPred (https://prediction.charite.de/ (accessed on 18 May 2023). All targets were standardized with their appropriate gene names in “Homo sapiens” using the UniProtKB database (https://www.uniprot.org/ (accessed on 18 May 2023).

#### 2.8.2. Identification of Inflammation- and Antioxidant-Related Target Genes

The Comparative Toxicogenomics Database (http://ctdbase.org/ (accessed on 18 May 2023), Online Mendelian Inheritance in Man database (https://www.omim.org/), and GeneCards database (https://www.genecards.org/ (accessed on 18 May 2023) were used to retrieve the target genes related to inflammation and antioxidant activity. After the compilation of all the obtained genes, duplicates were removed.

#### 2.8.3. Determination of Common Targets and Construction of Compound–Target and Protein–Protein Interaction Networks

All targets related to PFL compounds, inflammation, and antioxidant activity were uploaded to https://bioinformatics.psb.ugent.be/webtools/Venn/ (accessed on 19 May 2023) to obtain a Venn diagram. The overlapped genes were defined as common targets that demonstrate the interaction of inflammation- and antioxidant-related genes with PFL.

The relationships between the common targets and PFL compounds were constructed using Cytoscape 3.9.1 software. The compound–target network was then analyzed using the NetworkAnalyzer plugin. The top 10 components were defined as potent active compounds of PFL. To identify the key targets, the protein–protein interaction (PPI) of the most interacted genes in the compound–target network was collected from the STRING database with a required interaction score of 0.4 and the organism as *Homo sapiens*. The PPI network was then exported to Cytoscape and analyzed through the CytoHubba plugin to determine the top 10 hub genes according to the degree method.

#### 2.8.4. Gene Ontology (GO) and Kyoto Encyclopedia of Genes and Genomes (KEGG) Pathway Enrichment Analyses

The common targets were uploaded to DAVID v2021 (https://david.ncifcrf.gov/ (accessed on 19 May 2023) to analyze the enrichment of GO terms and KEGG pathways. The relevant GO terms and KEGG pathways were defined with a cutoff of *p* < 0.05. Among the GO terms, the top 10 biological process (BP), cellular component (CC), and molecular function (MF) GO terms were mapped; and, among the KEGG terms, the top 20 enriched KEGG pathways related to inflammation and antioxidant were mapped, using bioinformatics tools (http://www.bioinformatics.com.cn/ (accessed on 19 May 2023).

### 2.9. Computational Validation Based on Molecular Docking Analysis

The binding affinity between the active compounds in PFL and the key targets was analyzed using molecular docking simulations. The 3D structure of the potent active compounds acquired from PubChem as an SDF file was converted into PDBQT format using OpenBabel2.4.1. The crystal 3D structures of the key targets, composed of PTGS2 (PDB: 5IKQ), TLR4 (PDB: 3FXI), NFEL2 (PDB: 7X5E), PRKCD (PDB: 1YRK), NFKB1 (PDB: 1SCV), KEAP1 (PDB: 6TYP), and NR1l2 (PDB: 6P2B) were retrieved from Protein Databank (https://www.rcsb.org/). The initial structures were simplified via the deletion of water molecules and other small molecules using Discover Studio (version 2021). After the addition of polar hydrogens and Kollman charges using Autodock Tools 1.5.7, the protein structures were converted into PDBQT format. Molecular docking analysis was performed using Autodock Vina, with a grid box size of 50 × 50 × 50. The box center coordinates for each protein are provided in Table S1. The binding mode between the targets and the compounds was based on the binding energy value. Discovery studio software (Biovia DSV 2021) was further used to visualize the best pose with the lowest binding energy.

### 2.10. Statistical Analysis

The results were analyzed via one-way analysis of variance (ANOVA) followed by a Tukey post hoc test and expressed as mean ± standard deviation (SD). Values of * *p* < 0.05, ** *p* < 0.01, and *** *p* < 0.001 are considered a statistically significant difference.

## 3. Results and Discussion

### 3.1. Phytochemcal Profile of Lipophilic Fractions from P. fruticosa Leaves

The lipophilic compounds in PFLH and PFLD were tentatively identified using GC-MS analysis. Table 1 presents the 71 lipophilic compounds detected in the PFL. These compounds were categorized into eight groups, including fatty acids and esters (n = 14), furan derivative (n = 1), polyphenols (n = 9), hydrocarbons and oxygenated hydrocarbons (n = 4), polyacetylenes (n = 2), sterols (n = 2), terpenes (n = 28), tocols (n = 4), and other components (n = 7). As a member of the genus *Polyscias*, PFL shares similar chemical attributes with species in this genus [24].

Fatty acids and esters were indicated as the main components of PFL, accounting for 37.97% and 16.07% of PFLH and PFLD, respectively. Linolenic acid and 10-trans 12-cis-linoleic acid were the most abundant unsaturated fatty acids, followed by linoleic acid, linoelaidic acid, and 9,11-octadecadienoic acid. Unsaturated fatty acids were also found abundantly in species belonging to the family Araliaceae, as an example of *Panax ginseng* [25], and were recognized as exerting pleiotropic effects, such as antioxidant, anti-inflammatory, and cardioprotective properties [26,27]. PFL also contained a considerable amount of saturated fatty acids, such as palmitic acid and stearic acid, and esters, including 2-palmitoylglycerol, 2-linoleoylglycerol, and methyl linoleate.

A considerable proportion of terpenes were identified in PFL, which constituted 17.55% of PFLH and 19.07% of PFLD. PFL contained monoterpenes, diterpenes, triterpenes, and sesquiterpenes at different degrees. Phytol, neophytadiene, loliolide, (−)-isolongifolol methyl ether, (Z)-1,3-phytadiene, α-bergamotene, alismoxide, squalene, and 6,10,14-trimethylpentadecan-2-one were the most abundant terpene compounds in PFL. In addition, a moderate amount of sesquiterpenes, such as (−)-α-gurjunene, γ-elemene, (−)-germacrene D, (+)-γ-cadinene, ylangene, aromadendrene, caryophyllene oxide, and copaene, were also detected. In line with this study, a previous study showed diverse sesquiterpenes in the essential oil of *P. fruticosa* leaves [12].

Moreover, phytosterols, composed of stigmasta-7,16-dien-3-ol, (3β,5α) and stigmasterol, were detected in PFLH (8.89%) and PFLD (5.32%). Stigmasterol is one of the characteristic phytosterols detected in species of the genus *Polyscias*, while no study mentioned the presence of stigmasta-7,16-dien-3-ol, (3β,5α) in this genus. Additionally, polyacetylenes were also found in PFL, predominantly in PFLH. Falcarinol, an oxylipin-polyacetylene mainly found in plants belonging to the family Apiaceae, Asteraceae, and Araliaceae [28], and especially in the genus *Polyscias* [24], was the most dominant in PFL, followed by Hexadeca-5,7,9,11-tetrayne-1,16-diol. 

Several tocols, including α-, β-, and δ-tocopherols, were only detected in PFLH, whereas polyphenols, well-known antioxidant compounds, were only identified in PFLD. A significant amount of polyphenolic compounds, such as 2-methoxy-4-vinylphenol, 1′-hydroxyeugenol, sinapyl alcohol, 4-vinylphenol, vanillin, and 1,2-bis(4-hydroxy-3-methoxyphenyl)ethylene, were found in PFL, although no study has reported these compounds in the genus *Polyscias*.

### 3.2. Antioxidant and Anti-Inflammatory Mechanisms of Lipophilic Extracts from P. fruticosa Leaves Based on Network Pharmacology Analysis

#### 3.2.1. Network Construction and Analysis

**Figure 1:**
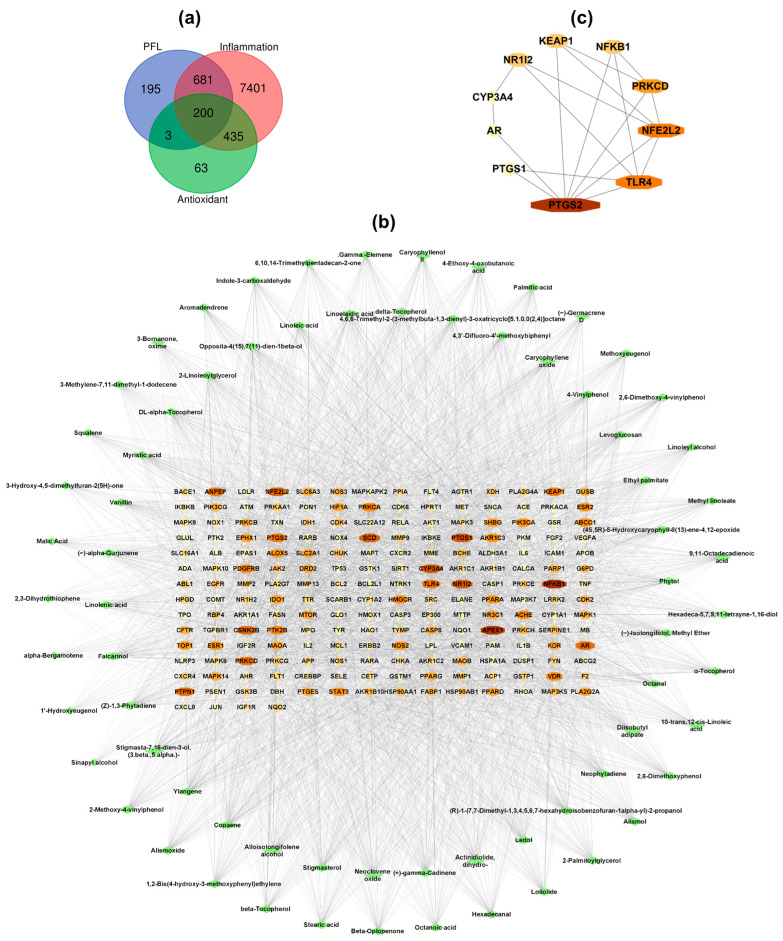
Network pharmacology analysis of PFL. (**a**) The Venn diagram of the genes related to PFL, inflammation, and antioxidant activity; (**b**) The compound–target network of PFL in modulating inflammation and the oxidative stress responses. The green diamonds represent the compounds, and the bigger the node, the higher the degree. The octagon represents the potential targets; the darker and the bigger the node, the higher the degree. (**c**) The protein–protein interactions between the top 10 genes. Nodes indicate proteins. Darker and bigger nodes suggest higher degree values. Lines represent protein–protein associations.

Network pharmacology analysis was performed to investigate the possible antioxidant and anti-inflammatory mechanisms of lipophilic extracts from PFL and the active compounds responsible for these biological activities. The putative targets of the selected 71 compounds were retrieved from the SuperPred and Swiss Target Prediction databases, which yielded a total of 1079 potential target genes. A total of 9418 target genes, including 8717 genes related to inflammation and 701 genes related to antioxidant activity, were acquired from GeneCards, CTD, and OMIM after the deletion of duplicates. These genes were intersected with the target genes of PFL compounds, and the Venn diagram showed 200 intersection genes, considered as common targets (Figure 1a).

To decipher the relationship between the bioactive compounds in PFL and common target genes, a compound–target network was built and analyzed using Cytoscape 3.9.1 software, and showed 271 nodes and 3327 edges (Figure 1b). The edge number suggested that one compound could affect multiple target genes, and multiple compounds could interact with a single target. The network analysis indicated that the average node degree was 46.85, and 43 compounds have degree values over the average degree value. The top 10 active compounds according to the degree value include falcarinol (degree = 62), (Z)-1,3-phytadiene (degree = 61), copaene (degree = 60), (3β,5α)-stigmasta-7,16-dien-3-ol, (degree = 60), ylangene (degree = 60), alloisolongifolene alcohol (degree = 57), (+)-γ-cadinene (degree = 56), neoclovene oxide (degree = 56), stigmasterol (degree = 56), and dihydroactinidiolide (degree = 55) (Table S2). Therefore, these compounds, mainly phytosterols and sesquiterpenes, were recognized as the potential compounds contributing to the antioxidant and anti-inflammatory activities of lipophilic extracts from PFL.

To aid a comprehensive understanding of the antioxidant and anti-inflammatory mechanisms of lipophilic extracts from PFL, the top 15 target genes from the compound-target network were introduced to the STRING database to analyze protein–protein interactions (PPIs) (Table S3). The PPI network was visualized via Cytoscape and analyzed using the degree method in the CytoHubba plugin to determine the top 10 Hub genes. As shown in Figure 1c, the top 10 Hub genes included prostaglandin-endoperoxide synthase 2 (PTGS2), toll-like receptor 4 (TLR4), nuclear factor erythroid 2-like 2 (NFE2L2), protein kinase C delta (PRKCD), Kelch-like ECH associated protein 1 (KEAP1), nuclear factor kappa B subunit 1 (NFKB1), nuclear receptor subfamily 1 group l member 2 (NR1I2), prostaglandin-endoperoxide synthase 1 (PTGS1), androgen receptor (AR), and cytochrome P450 3A4 (CYP3A4). In general, lipophilic extracts from PFL may exhibit antioxidant and anti-inflammatory abilities by regulating these core genes.

The PTGS group, known as COX, comprises major enzymes responsible for converting arachidonic acid to prostaglandins, which are implicated in multiple physiological and pathological processes [29]. PTGS1 (COX-1) is a housekeeping gene that ensures the production of prostaglandins necessary for normal physiological functions, while PTGS2 (COX-2) is an inducible isoform induced by diverse stimuli, such as endotoxins, cytokines, and growth factors, and its overexpression is closely associated with acute and chronic inflammation [29,30]. The release of inflammatory factors at the inflammation site is the result of the activation of several upstream transcription factors, including nuclear factor-kappa B (NFKB). NFKB is implicated in almost all aspects of immunoinflammatory responses. In addition to including a broad range of pro-inflammatory cytokines, chemokines, and mediators, NFKB participates in regulating the activation and differentiation of inflammatory T cells, as well as the activation of inflammasomes [31]. In addition, in response to the inflammatory stimuli, namely bacterial endotoxins (e.g., LPS), TLR4, a well-studied pattern recognition receptor, triggers downstream signaling cascades, subsequently resulting in NFKB activation, a hallmark of inflammatory diseases [32].

NFE2L2, known as Nrf2, is a redox transcription factor that vitally contributes to cellular redox homeostasis by controlling the expression of phase II detoxification and antioxidant enzymes, namely heme oxygenase-1 (HO-1), NQO1 NAD(P)H quinone dehydrogenase 1 (NQO1), catalase (CAT), glutathione peroxidase (GPx), and superoxide dismutase (SOD). As a negative regulator, KEAP1 sequesters NFE2L2 in the cytosol under physiological conditions, consequently leading to the ubiquitination and degradation of NFE2L2. However, in the presence of electrophilic/oxidative stress or stimuli, NFE2L2 can split from KEAP1 and translocate into the nucleus to induce the expression of antioxidant response element-mediated genes [33]. NFE2L2 knockout mice exhibited a significant reduction in their levels of phase II enzymes and sensitivity to carcinogens; the NFE2L2 deletion exacerbates oxidative-stress-mediated pathological conditions [1,34].

NR1I2, also designated as pregnane X receptor (PXR), is a nuclear receptor exerting pleiotropic functions, including xenobiotic metabolism and immunomodulation. PXR plays a vital role in cellular defense against oxidative stress by regulating phase I and phase II enzymes, such as cytochrome P450 (e.g., CYP3A4) and glutathione S- transferase [35]. Among phase I enzymes, CYP3A4, which is responsible for the metabolism of xenobiotics and facilitating their elimination, appears to cytoprotectively act more than others [36]. On the other hand, PXR also plays an anti-inflammatory role by negatively regulating the TLR4/NF-κB pathway, suggesting a potent target for the management of inflammation-related diseases [35,37].

PRKCD, belonging to the family of serine/threonine protein kinase C, is involved in multiple physiological events, such as cell proliferation, apoptosis, angiogenesis, and the immune response. PRKCD-mediated p62 phosphorylation causes the degradation of KEAP1, leading to the stabilization and accumulation of NFE2L2 in the nucleus. Furthermore, androgen receptors (ARs), androgen-dependent transcription factors, play a crucial role in the physiological functioning of the reproductive system [38]. The dysregulation of AR causes impairment to the reproductive function and is closely associated with the onset of liver, bladder, breast, and prostate cancer [39,40]. Accumulating evidence has shown that AR overactivation induces the aberrant production of COX-2 in periovulatory granulosa cells and urothelial carcinoma cells [38,40].

Overall, these findings suggest that the antioxidant and anti-inflammatory potentials of PFL may be achieved through the regulation of core target genes, including TLR4, NFKB1, PTGS1, PTGS2, AR, NFE2L2, NR1l2, PRKCD, CYP3A4, and KEAP1.

#### 3.2.2. Enrichment Analysis of Gene Ontology (GO) and Kyoto Encyclopedia of Genes and Genomes (KEGG) Pathways

**Figure 2:**
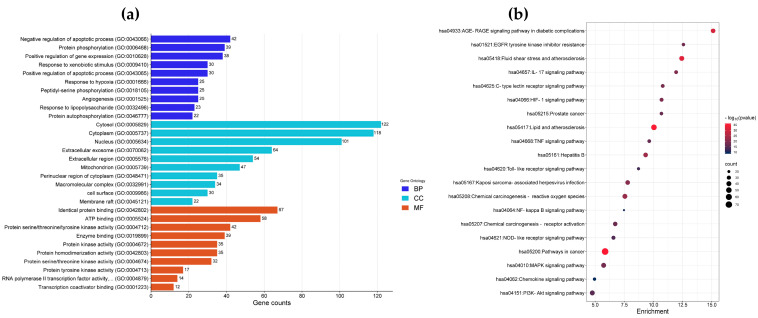
GO and KEGG pathway enrichment analyses. (**a**) Bar chart of the top 10 GO biological processes (BPs), cellular components (CCs), and molecular functions (MFs); (**b**) bubble chart of the top 20 KEGG enriched pathways.

To determine the functions of lipophilic extracts from PFL in regulating inflammatory and oxidative responses, GO and KEGG enrichment analyses of common targets were analyzed using DAVID bioinformatic resources. The GO analysis showed a total of 969 GO items (*p* < 0.05), including 704 biological process (BP) terms, 92 cell component (CC) terms, and 173 molecular function (MF) terms. The top 10 enriched BP, CC, and MF GO terms are presented in Figure 2a. The main BP terms were negative/positive regulation of apoptotic processes, response to xenobiotic stimuli, response to hypoxia, angiogenesis, and response to lipopolysaccharide. The main CC terms were cytosol, cytoplasm, nucleus, extracellular exosome, mitochondrion, cell surface, and membrane raft. The MF terms were mainly related to identical protein binding, ATP binding, protein serine/threonine/tyrosine kinase activity, enzyme binding, protein kinase activity, and transcription coactivator binding. Moreover, 182 KEGG pathways were significantly enriched (*p* < 0.05); among them, bioactive compounds of PFL might mainly affect the AGE–RAGE signaling pathway in diabetic complications, EGFR tyrosine kinase inhibitor resistance, fluid shear stress and atherosclerosis, the IL-17 signaling pathway, the HIF-1 signaling pathway, the TNF signaling pathway, the toll-like receptor (TLR) signaling pathway, chemical carcinogenesis–reactive oxygen species, the NF-kappa B signaling pathway, the NOD-like receptor (NLR) signaling pathway, the MAPK signaling pathway, and the PI3K-Akt signaling pathway. Figure 2b shows the top 20 KEGG enrichment pathways of the common targets.

The NF-κB signaling pathway plays a key role in both the innate and adaptive immune response. Various upstream signaling pathways, such as the AGE–RAGE, IL-17, TNF, TLR, PI3K-Akt, MAPK, and NLR signaling pathways, can trigger NF-κB activation, leading to the expression of inflammation-related genes [31]. The TLR signal is one of the most extensively studied pathogen-associated molecular pattern (PAMP) recognition receptors. By recognizing PAMPs, TLR activates diverse cellular signaling cascades that converge and stimulate NF-κB activity. Upon activation, NF-κB drives the expression of pro-inflammatory cytokines, such as pro-IL-1β and pro-IL-18, which are subsequently converted to their mature forms via the oligomerized NLRP3 inflammasome, a well-studied NLR signaling pathway [32]. In addition, the IL-17 and TNF signaling pathways are implicated in the onset and progression of multiple inflammation-related diseases, such as sepsis, rheumatoid arthritis, inflammatory bowel disease, and cancer [41,42]. The binding of advanced glycation end-products (AGEs) and their receptors (RAGEs) can provoke various downstream signaling pathways, such as the PI3K-Akt, MAPK, and NF-κB signaling pathways, which promote ROS formation, the generation of inflammatory factors, and mitochondrial dysfunction [43]. The AGE–RAGE signaling pathway is involved in the onset and progression of several inflammation and oxidative-stress-related diseases [44].

In addition, mitogen-activated protein kinases (MAPKs) and phosphoinositide 3-kinase (PI3K)–Akt are well-known signal transduction pathways that mediate diverse physiological processes, such as proliferation, growth, differentiation, and apoptosis. The activation of the PI3K–Akt signaling pathway has been found to be closely associated with osteoarthritis by inducing the activation of NF-κB signaling and the expression of inflammatory cytokines [45]. The inhibition of the PI3K–Akt signaling pathway reduces levels of NF-κB-mediated inflammatory factors and, therefore, suggests a potential therapy approach for osteoarthritis [46]. In addition, the MAPK signaling pathway is involved in both inflammation and cellular antioxidant defense mechanisms through activating redox transcription factors, namely NF-κB and Nrf2 [47]. Moreover, the hypoxia-inducible factor 1 (HIF-1) signaling pathway plays an important role in the regulation of inflammation, hypoxia, and oxidative stress. The HIF-1 signaling pathway is involved in inflammatory functions as it drives the expression of pro-inflammatory genes [16]. In addition, the HIF-1 signaling pathway is also associated with the cellular antioxidant defense against oxidative stress, through enhancing the levels of Nrf2 and phase II antioxidant enzymes [48].

As the enriched pathways are mostly related to inflammation and oxidative stress, lipophilic extracts of *P. fruticosa* leaves may alleviate inflammatory and oxidative responses by regulating these pathways.

### 3.3. Molecular Docking Validation

**Figure 3:**
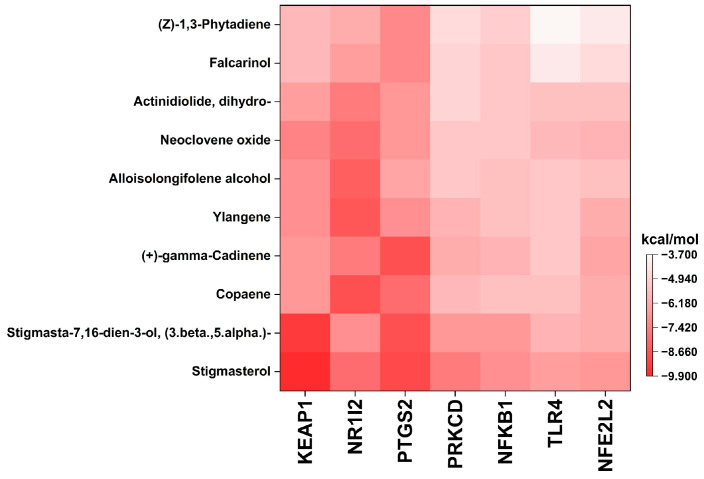
Heatmap representing the binding free energies between the top 10 active compounds and the most active targets related to PFL. The colored rectangles indicate the binding energy (kcal/mol), and the dark red color shows a lower binding free energy.

**Figure 4:**
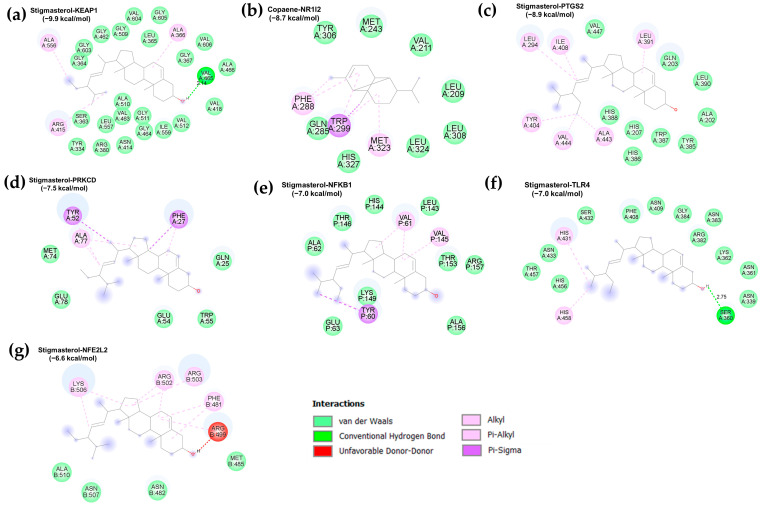
A representation of the binding modes of PFL active compounds and the top hub genes with the lowest binding free energy. (**a**) Stigmasterol-KEAP1 (−9.9 kcal/mol); (**b**) copaene-NR1l2 (−8.7 kcal/mol); (**c**) stigmasterol-PTGS2 (− 8.9 kcal/mol); (**d**) stigmasterol-PRKCD (−7.5 kcal/mol); (**e**) stigmasterol-NFKB1 (−7.0 kcal/mol); (**f**) stigmasterol-TLR4 (−6.4 kcal/mol); and (**g**) cycloartenol-NFE2L2 (−6.6 kcal/mol).

The interactions between the top 10 active compounds of PFL and key targets [namely PTGS2 (PDB: 5IKQ), TLR4 (PDB: 3FXI), NFEL2 (PDB: 7X5E), PRKCD (PDB: 1YRK), NFKB1 (PDB: 1SCV), KEAP1 (PDB: 6TYP), and NR1l2 (PDB: 6P2B)] were elucidated using molecular docking analysis. A negative binding energy indicates a spontaneous binding between the receptor and the ligand, and a lower binding energy reflects a stable binding conformation [17]. In general, a free binding energy below −5.0 kcal/mol was regarded as a good binding conformation between a ligand and a receptor. A heatmap indicates the binding energies of the interactions between the active compounds and target proteins (Figure 3 and Table S4). The results showed that the active compounds of the PFL and Hub genes had binding energies ranging from −9.9 kcal/mol to −3.7 kcal/mol, and 63 out of 70 docking results had binding energies below −5.0 kcal/mol, and 20 results were below −7.0 kcal/mol. The binding poses of the representative docking results are illustrated in Figure 4.

Stigmasterol and (3β,5α)-stigmasta-7,16-dien-3-ol exhibited strong binding activities and stable conformations with KEAP1 (−9.9 and −9.4 kcal/mol, respectively) and PTGS2 (−8.9 and −8.8 kcal/mol, respectively). In addition, stigmasterol showed a high binding affinity with NR1l2 (−7.9 kcal/mol), PRKCD (−7.6 kcal/mol), and NFKB1 (−7.0 kcal/mol). These compounds belong to the phytosterols that are known to possess multiple health-promoting effects, such as antioxidant, anti-inflammatory, anti-bacterial, and immunomodulatory properties [49]. Accumulating evidence indicates the role of the hydroxyl group on the side chain of stigmasterol in its antioxidant capacity [50]. Additionally, in vitro and in vivo studies have demonstrated the anti-inflammatory capacity of stigmasterol through its suppression of the expression of NF-κB-mediated pro-inflammatory genes and mediators [51,52]. The biological potential of stigmasterol was confirmed in a cerebral ischemia/reperfusion injury model, which indicated that stigmasterol attenuated inflammation by downregulating the NF-κB pathway and improved the antioxidant defense by upregulating the Nrf2 pathway [53].

Copaene, (+)-gamma-cadinene, and ylangene had robust binding interactions with NR1l2 (−8.7, −8.7, and −8.6 kcal/mol, respectively) and PTGS2 (−8.0, −8.0, and −7.0 kcal/mol, respectively). These compounds belong to sesquiterpenes, one of the largest groups of phytochemicals, and offer health-protective effects against metabolic disorders and cancer [54]. The copaene-rich fraction from *Annona reticulata* L. bark has been demonstrated to alleviate carrageenan-induced paw inflammation in rats [55]. *Lippia graveolens* and *Lippia palmeri* extracts containing gamma-cadinene exert antioxidant and anti-inflammatory abilities by reducing the levels of NO, ROS, and COX-2 in LPS-treated RAW 264.7 cells [56]. In addition, α-ylangene-rich essential oil from *Schisandra chinensis* exhibits antioxidant capacity by mitigating ROS production in UVB-exposed HaCaT cells and anti-inflammatory activity by reducing NO and iNOS levels through downregulating NF-κB activity in LPS-treated RAW 264.7 cells [57].

Alloisolongifolene alcohol and neoclovene oxide showed good binding capacities with KEAP1 (−7.0 and −7.3 kcal/mol, respectively) and NR1l2 (−8.3 and −8.0 kcal/mol, respectively). Dihydroactinidiolide showed a strong affinity with NR1l2 (−7.5 kcal/mol), while (Z)-1,3-phytadiene and falcarinol had robust conformations with PTGS2 (−7.1 and −7.1 kcal/mol). Falcarinol, a polyacetylenic oxylipin, offers several biological functions, such as anti-inflammatory, anti-diabetic, cytotoxic, chemopreventive, and anti-neoplastic effects [58,59]. In vitro and in vivo studies have documented the anti-inflammatory ability of falcarinol via the downregulation of NF-κB activity and the expression of inflammatory factors [58,59].

### 3.4. Chemical-Based Antioxidant Activity of Lipophilic Extracts from P. fruticosa Leaves

An in vitro system is a widely used method for evaluating the antioxidant capacity of natural compounds. Various chemical mechanisms, such as free radical scavenging through electron donation and hydrogen radical transfer, and neutralization through redox reactions with various metal ions, can be assessed [60]. Thus, in this study, five radical scavenging activity assays (DPPH^•^, ABTS^•+^, O_2_^•−^, OH^•^, NO^•^, and H_2_O_2_) and three reducing power assays (FRAP, PFRAP, and TAC) were conducted to depict the antioxidant activities of lipophilic fractions from PFL. The radical scavenging activities of lipophilic fractions from PFL are presented in Table 2.

The scavenging of DPPH and ABTS^+^ radicals based on the donation of electrons and hydrogen atoms by antioxidant compounds is the most commonly used procedure to estimate the antioxidant activity of both hydrophilic and hydrophobic components [60]. Table 2 shows that both PFLH and PFLD were more effective in eliminating ABTS^+^ radicals compared to DPPH radicals. In addition, the DPPH and ABTS^+^ radical scavenging activities of PFLD (IC50 = 0.73 ± 0.02 and 0.14 ± 0.02, respectively) were better than those of PFLH (IC50 = 1.22 ± 0.03 and 0.67 ± 0.06, respectively).

The superoxide anion radical is one the most common in the human body and is involved in the creation of other oxidants. Hydrogen peroxide is produced via the spontaneous dismutation of the superoxide radical. It easily penetrates biological membranes and is implicated in the generation of a highly reactive hydroxyl radical via Haber–Weiss and Fenton reactions. Although the superoxide radical and hydrogen peroxide are weak reactive species, they also cause damage to cellular components and the aging process [61]. Herein, the IC_50_ values for the hydrogen peroxide scavenging activities of PFLH and PFLD were 0.26 ± 0.03 and 0.31 ± 0.04 mg/mL, respectively. PFLH (IC_50_ = 0.11 ± 0.04 mg/mL) was more effective in superoxide radical scavenging than PFLD (IC_50_ = 1.11 ± 0.35 mg/mL).

The hydroxyl radical, the most powerful ROS, can rapidly react with biomolecules, namely lipids, proteins, and DNA, resulting in membrane peroxidative damage, protein oxidative damage, and mutants. Hydroxyl radicals can be generated from the interaction of hydrogen peroxide with transitional metal ions, the breakdown of water via ion radiation, or the photolytic decomposition of alkyl hydroperoxides [61,62]. This study showed that PFLD (IC_50_ = 0.27 ± 0.06 mg/mL) exhibited a greater hydroxyl radical scavenging activity than PFLH (IC_50_ = 4.91 ± 2.25 mg/mL), which can possibly be attributed to the predominance of phenolic compounds in PFLD.

Nitric oxide is an abundant reactive molecule involved in multiple physiological and pathological processes. Due to its solubility in both hydrophilic and hydrophobic environments, nitric oxide can easily permeate through the plasma membrane and react with superoxide radicals to form peroxynitrite, a highly reactive species that causes cellular oxidative damage [63]. Here, compared to PFLH (IC_50_ = 6.01 ± 0.57 mg/mL), PFLD (IC_50_ = 0.39 ± 0.05 mg/mL) was much more effective in eliminating nitric oxide radicals.

The reducing power is important in evaluating the antioxidant capacity of extracts/phytochemicals based on their abilities to reduce metal ions by donating an electron [60]. This study indicated that PFLD exerted a strong reducing potential, as evidenced by the FRAP, PFRAP, and TAC assays, compared to PFLH.

Overall, these findings suggest that the lipophilic fractions from PFL exert strong antioxidant capacities through their radical scavenging ability and reducing potential. The higher antioxidant activity of PFLD may be ascribed to the predominance of bioactive compounds, such as polyphenols and terpenes.

### 3.5. Antioxidant Activity of Lipophilic Extracts from P. fruticosa Leaves in LPS-Stimulated RAW 264.7 Cells

The cells are equipped with a complex endogenous antioxidant defense system, including a wide range of antioxidant enzymes and nonenzymatic molecules, to maintain cellular redox homeostasis. However, these systems can be depleted under the condition of oxidative stress, facilitating the pathological state [61,63]. Therefore, the supplementation of dietary antioxidants is an effective method for protecting against oxidative damage and, thereby, preventing the development of diseases.

**Figure 5:**
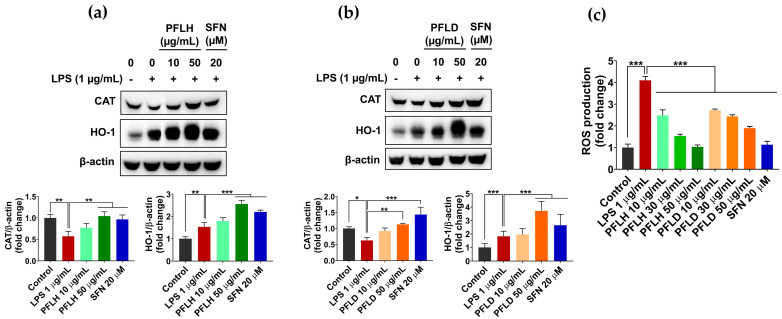
The antioxidant activity of lipophilic extracts from PFL in LPS-treated RAW 264.7 cells. The cells were pretreated with PFLH or PFLD for 1 h before LPS (1 μg/mL) challenge for 6 h or 12 h. (**a**,**b**) The protein expression of CAT and HO-1 after 12 h of treatment. (**c**) The ROS accumulation after 6 h of treatment. The results are presented as the mean ± SD. Values of * *p* < 0.05, ** *p* < 0.01, and *** *p* < 0.001 are considered a statistically significant difference. PFLH: *P. fruticosa* leaf hexane extract; PFLD: *P. fruticosa* leaf dichloromethane extract; SFN: sulforaphane; LPS: lipopolysaccharide.

Primary antioxidant enzymes, such as catalase (CAT), glutathione peroxidase, and superoxide dismutase provide vital protection against oxidative stress by decomposing and nullifying radicals/oxidants before they can cause considerable damage to cellular biomolecules [64]. In this study, LPS challenge significantly reduced CAT expression in the RAW 264.7 cells, compared to the untreated cells. However, the pretreatment of PFLH or PFLD restored the level of this protein, with abilities comparable to sulforaphane (SFN, a positive control) (Figure 5a,b).

As a member of the cellular antioxidant defense system, the phase II antioxidant enzyme heme oxygenase-1 (HO-1) also actively participates in the cellular defense mechanism against a massive oxidative attack. HO-1 is involved in the oxidation of heme to ferrous ions, carbon monoxide (CO), and biliverdin, with the latter further converted to bilirubin. Bilirubin is a powerful antioxidant that can suppress the activity of NADPH oxidase and, thereby, prevent ROS formation, while CO has important physiological functions, such as anti-inflammatory, anti-apoptosis, and vasodilatory properties. Heme catabolism by HO-1 contributes to preventing the cellular accumulation of free heme, which mediates the generation of highly reactive oxidants via the Fenton reaction [65]. In addition to limiting the expression of pro-inflammatory genes through the downregulation of NF-κB activation, HO-1 is implicated in regulating the proliferation and differentiation of inflammatory T cells, as well as the functions of macrophages and dendritic cells [65,66]. Thus, the inductive effects of PFLH and PFLD on HO-1 expression in LPS-treated cells were examined. As shown in Figure 5a,b, the pretreatment of PFLH or PFLD considerably enhanced the HO-1 level in LPS-treated macrophages. Consequently, the LPS-triggered ROS accumulation in the macrophages was markedly attenuated in the presence of PFLH or PFLD (Figure 5c).

Taken together, these findings propose that lipophilic fractions from PFL exert a potent antioxidant activity by upregulating the expression of cellular antioxidant enzymes, in addition to their oxidant scavenging potential.

### 3.6. Anti-Inflammatory Activity of Lipophilic Extracts from P. fruticosa Leaves in LPS-Treated RAW 264.7 Cells

Inflammation, a defense mechanism of the human body against bacterial/viral infection and injuries, is mediated by various non-immune and immune cells [3]. Among these, macrophages play a crucial role in all stages of the inflammatory process. In response to inflammatory stimuli, including bacterial endotoxins (e.g., LPS) and oxidative stress, activated macrophages release a broad spectrum of pro-inflammatory mediators, cytokines, chemokines, and growth factors. The overproduction of these factors results in acute/chronic inflammation, consequently causing the initiation and progression of multiple diseases, such as autoimmune diseases, inflammatory bowel diseases, and arthritis [67]. The crosstalk between oxidative stress and inflammation implies that oxidative stress can trigger the inflammatory response; conversely, inflammation also promotes the formation of oxidants, aggravating the inflammatory conditions [5,6]. Therefore, the management of inflammation via regulating the generation of inflammatory factors has an important contribution to preventing the development of diseases.

**Figure 6:**
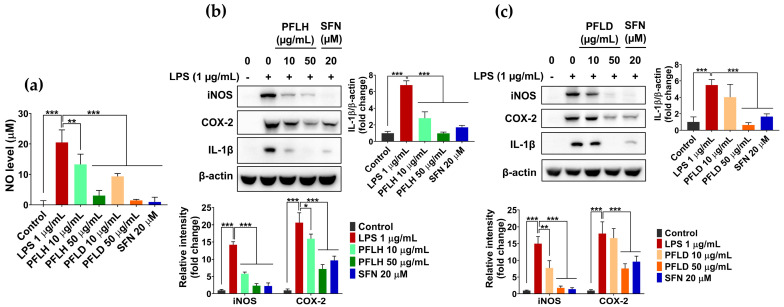
The anti-inflammatory activity of lipophilic extracts from PFL in LPS-treated RAW 264.7 cells. The cells were pretreated with PFLH or PFLD for 1 h before LPS (1 μg/mL) challenge for 12 h. (**a**) NO production. (**b**,**c**) The protein expression of iNOS, COX-2, and IL-1β. The results are presented as the mean ± SD. Values of * *p* < 0.05, ** *p* < 0.01, and *** *p* < 0.001 are considered a statistically significant difference. PFLH: *P. fruticosa* leaf hexane extract; PFLD: *P. fruticosa* leaf dichloromethane extract; SFN: sulforaphane; LPS: lipopolysaccharide.

In this study, the anti-inflammatory potential of lipophilic extracts from PFL was verified using LPS-treated RAW 264.7 cells. As shown in Figure 6a, LPS challenge remarkably increased the NO level in the macrophage culture medium; in contrast, the pretreatment of PFLH or PFLD considerably reduced the NO level, suggesting their anti-inflammatory potential. To further elucidate the role of PFLH and PFLD in managing inflammation, the effects of PFLH and PFLD on the expression levels of inflammatory factors were inspected in LPS-treated RAW 264.7 cells. LPS treatment significantly enhanced the levels of iNOS and COX-2, compared to the untreated control. However, the LPS-induced levels of these enzymes were significantly alleviated in the presence of PFLH or PFLD (Figure 6b,c). In addition, LPS challenge substantially upregulated IL-1β expression in macrophages; this effect was considerably reduced with the pretreatment of PFLH or PFLD (Figure 6b,c). Taken together, these observations suggest that the lipophilic fractions from PFL exert anti-inflammatory properties, in addition to their antioxidant abilities.

## 4. Conclusions

This study provides insights into the antioxidant and anti-inflammatory mechanisms of lipophilic extracts from *P. fruticosa* leaves, using an integrative approach of network pharmacology analysis and in silico and in vitro verification. Network pharmacology and molecular docking analyses revealed that (3β,5α)-stigmasta-7,16-dien-3-ol, (+)-γ-cadinene, copaene, ylangene, alloisolongifolene alcohol, and neoclovene oxide are the key active lipophilic compounds in PFL that are responsible for its biological properties. In addition, these compounds mainly affected core targets, including PTGS2, TLR4, NFE2L2, PRKCD, KEAP1, NFKB1, NR1l2, PTGS1, AR, and CYP3A4, mostly enriched in the AGE–RAGE, IL-17, TNF, TLR, NF-κB, NLR, MAPK, PI3K-Akt, and HIF-1 signaling pathways. Furthermore, lipophilic extracts from PFL were found to exhibit powerful antioxidant and anti-inflammatory capacities, as evident in the in vitro chemical- and cell-based experimental verifications. In conclusion, this study could provide useful information for developing nutraceuticals and functional foods from the lipophilic components of PFL, with antioxidant and anti-inflammatory potential.

## Figures and Tables

**Table 1:** The chemical compositions of lipophilic extracts from *P. fruticosa* leaves, detected via GC-MS.

Group	Retention Time	Compound	Molecular Formula	Peak Area (%)	
				PFLH	PFLD
Fatty acids and esters (n = 14)	22.088	Octanoic acid	C_8_H_16_O_2_	0.51 ± 0.05	0.97 ± 0.41
	35.66	Diisobutyl adipate	C_14_H_26_O_4_	0.54 ± 0.15	0.52 ± 0.24
	37.298	Myristic acid	C_14_H_28_O_2_	0.40 ± 0.01	-
	41.518	Palmitic acid	C_16_H_32_O_2_	10.72 ± 0.05	5.04 ± 1.95
	42.154	Ethyl palmitate	C_18_H_36_O_2_	0.45 ± 0.17	-
	44.102	Methyl linoleate	C_19_H_34_O_2_	0.15 ± 0.01	-
	44.751	10-trans,12-cis-Linoleic acid	C_18_H_32_O_2_	8.15 ± 0.12	2.85 ± 1.03
	44.901	Linolenic acid	C_18_H_30_O_2_	7.73 ± 0.04	2.56 ± 0.95
	44.988	Linoleic acid	C_18_H_32_O_2_	-	0.52 ± 0.19
	45.109	Linoelaidic acid	C_18_H_32_O_2_	0.98 ± 0.01	-
	45.385	Stearic acid	C_18_H_36_O_2_	2.28 ± 1.08	1.1 ± 0.43
	45.706	9,11-Octadecadienoic acid	C_18_H_32_O_2_	0.24 ± 0.09	-
	51.148	2-Palmitoylglycerol	C_19_H_38_O_4_	2.82 ± 0.17	2.51 ± 0.25
	53.871	2-Linoleoylglycerol	C_21_H_38_O_4_	2.90 ± 0.24	-
Furan derivatives (n = 1)	19.981	3-Hydroxy-4,5-dimethylfuran-2(5H)-one	C_6_H_8_O_3_	-	0.95 ± 0.03
Hydrocarbons and oxygenated hydrocarbons (n = 4)	16.99	Octanal	C_8_H_16_O	0.18 ± 0.01	1.49 ± 0.12
	36.497	Hexadecanal	C_16_H_32_O	0.67 ± 0.03	-
	39.961	3-Methylene-7,11-dimethyl-1-dodecene	C_15_H_28_	-	1.74 ± 0.02
	53.857	Linoleyl alcohol	C_18_H_34_O	-	1.40 ± 0.01
Polyphenols (n = 9)	23.663	4-Vinylphenol	C_8_H_8_O	-	0.47 ± 0.01
	26.585	2-Methoxy-4-vinylphenol	C_9_H_10_O_2_	-	2.01 ± 0.87
	27.498	2,6-Dimethoxyphenol	C_8_H_10_O_3_	-	0.15 ± 0.05
	28.919	Vanillin	C_8_H_8_O_3_	-	0.39 ± 0.19
	32.967	2,6-Dimethoxy-4-vinylphenol	C_10_H_12_O_3_	-	0.31 ± 0.11
	36.157	4-Allyl-2,6-dimethoxyphenol	C_11_H_14_O_3_	-	0.21 ± 0.02
	37.07	1′-Hydroxyeugenol	C_10_H_12_O_3_	-	1.79 ± 0.67
	42.173	Sinapyl alcohol	C_11_H_14_O_4_	-	0.96 ± 0.34
	53.517	1,2-Bis(4-hydroxy-3-methoxyphenyl)ethylene	C_16_H_16_O_4_	-	0.34 ± 0.04
Polyacetylenes (n = 2)	43.102	Falcarinol	C_17_H_24_O	7.36 ± 0.24	-
	48.283	Hexadeca-5,7,9,11-tetrayne-1,16-diol	C_16_H_18_O_2_	-	0.94 ± 0.01
Sterol derivatives (n = 2)	64.481	Stigmasterol	C_29_H_48_O	6.53 ± 0.07	4.00 ± 1.47
	66.033	(3beta,5alpha)-Stigmasta-7,16-dien-3-ol	C_29_H_48_O	2.36 ± 0.09	1.32 ± 0.46
Terpenes (n = 28)	28.459	Ylangene	C_15_H_24_	0.06 ± 0.02	-
	28.628	Copaene	C_15_H_24_	0.04 ± 0.01	-
	29.935	gamma-Elemene	C_15_H_24_	0.12 ± 0.01	-
	31.2	alpha-Bergamotene	C_15_H_24_	1.65 ± 0.04	-
	31.431	(−)-Germacrene D	C_15_H_24_	0.47 ± 0.08	-
	32.153	(+)-gamma-Cadinene	C_15_H_24_	0.07 ± 0.01	-
	32.409	(−)-alpha-Gurjunene	C_15_H_24_	0.07 ± 0.01	-
	32.633	Actinidiolide, dihydro-	C_11_H_16_O_2_	0.32 ± 0.01	0.23 ± 0.01
	33.998	Caryophyllene oxide	C_15_H_24_O	0.18 ± 0.02	-
	34.885	Alismol	C_15_H_24_O	0.82 ± 0.01	-
	35.304	Opposita-4(15),7(11)-dien-1beta-ol	C_15_H_24_O	0.48 ± 0.04	-
	35.513	Alloisolongifolene alcohol	C_15_H_24_O	0.47 ± 0.01	-
	36.954	Aromadendrene	C_15_H_24_	0.10 ± 0.01	-
	37.302	3-Bornanone, oxime	C_10_H_17_NO	-	1.53 ± 0.14
	37.683	(4S,5R)-5-Hydroxycaryophyll-8(13)-ene-4,12-epoxide	C_15_H_24_O_2_	0.26 ± 0.01	
	37.96	Loliolide	C_11_H_16_O_3_	-	2.23 ± 1.09
	38.109	Alismoxide	C_15_H_26_O_2_	-	1.14 ± 0.57
	39.075	Neophytadiene	C_20_H_38_	3.24 ± 0.01	3.87 ± 1.47
	39.18	6,10,14-Trimethylpentadecan-2-one	C_18_H_36_O	1.02 ± 0.07	-
	39.963	(Z)-1,3-Phytadiene	C_20_H_38_	1.03 ± 0.01	-
	40.2	beta-Oplopenone	C_15_H_24_O	-	1.93 ± 1.03
	40.792	Neoclovene oxide	C_15_H_24_O	-	0.85 ± 0.01
	41.646	(−)-Isolongifolol methyl ether	C_16_H_28_O	-	2.70 ± 0.21
	41.958	Ledol	C_15_H_26_O	-	0.72 ± 0.01
	42.218	(R)-1-(7,7-Dimethyl-1,3,4,5,6,7-hexahydroisobenzofuran-1alpha-yl)-2-propanol	C_13_H_22_O_2_	-	1.00 ± 0.01
	43.066	Caryophyllenol II	C_15_H_24_O	-	0.79 ± 0.30
	44.4	Phytol	C_20_H_40_O	5.49 ± 2.65	2.08 ± 1.00
	55.615	Squalene	C_30_H_50_	1.66 ± 0.45	-
Tocols (n = 4)	57.714	delta-Tocopherol	C_27_H_46_O_2_	0.62 ± 0.08	-
	59.26	beta-Tocopherol	C_28_H_48_O_2_	0.98 ± 0.09	-
	61.126	DL-alpha-Tocopherol	C_29_H_50_O_2_	0.54 ± 0.25	-
	69.196	alpha-Tocopherol	C_29_H_50_O_2_	0.96 ± 0.01	-
Others (n = 7)	21.773	4-Ethoxy-4-oxobutanoic acid	C_6_H_10_O_4_	-	1.05 ± 0.40
	23.134	2,3-Dihydrothiophene	C_4_H_6_S	-	0.54 ± 0.04
	24.921	Malic acid	C_4_H_6_O_5_	-	1.29 ± 0.01
	31.125	Levoglucosan	C_6_H_10_O_5_	-	0.72 ± 0.01
	36.4	4,3′-Difluoro-4′-methoxybiphenyl	C_13_H_10_F_2_O	0.67 ± 0.04	-
	38.655	Indole-3-carboxaldehyde	C_9_H_7_NO	-	0.99 ± 0.01
	40.602	4,6,6-Trimethyl-2-(3-methylbuta-1,3-dienyl)-3-oxatricyclo [5.1.0.0(2,4)]octane	C_15_H_22_O	-	1.11 ± 0.43

**Table 2:** The cell-free antioxidant activity of lipophilic extracts from *P. fruticosa* leaves.

Antioxidant Activity	PFLH	PFLD
DPPH (IC_50_, mg/mL)	1.22 ± 0.03	0.73 ± 0.02 *
ABTS^+^ (IC_50_, mg/mL)	0.67 ± 0.06	0.14 ± 0.02 **
Superoxide radical (IC_50_, mg/mL)	0.11 ± 0.04	1.11 ± 0.35 **
Hydrogen peroxide (IC_50_, mg/mL)	0.26 ± 0.03	0.31 ± 0.04
Hydroxyl radical (IC_50_, mg/mL)	4.91 ± 2.25	0.27 ± 0.06 **
Nitric oxide (IC_50_, mg/mL)	6.01 ± 1.57	0.39 ± 0.05 *
FRAP (mg TE/g dried extract)	17.89 ± 0.25	29.55 ± 0.44 ***
PFRAP (mg TE/g dried extract)	22.18 ± 1.03	32.14 ± 2.68 **
TAC (mg TE/g dried extract)	9.54 ± 0.17	130.59 ± 1.75 ***

Values of * *p* < 0.05, ** *p* < 0.01, and *** *p* < 0.001 are considered statistically significant differences. PFLH: *P. fruticosa* leaf hexane extract; PFLD: *P. fruticosa* leaf dichloromethane extract; FRAP: ferric reducing antioxidant power; PFRAP: potassium ferricyanide reducing antioxidant power; TAC: total antioxidant capacity.

## Data Availability

The data, analytic methods, and study materials that support the findings of this study are available from the corresponding author upon reasonable request.

## Supplementary Materials

The following supporting information can be downloaded at: https://www.mdpi.com/article/10.3390/foods12193643/s1, Table S1: The PDB ID and the box center coordinates for each protein. Table S2: Top 10 active compounds in PFL obtained from PFL compounds-target network. Table S3: Top 15 target genes obtained from PFL compounds-target network. Table S4: Molecular docking results of key active compounds in PFL and the hub genes.
