# Supplementary material for: Antioxidant and Anti-Inflammatory Mechanisms of Lipophilic Fractions from Polyscias fruticosa Leaves Based on Network Pharmacology, In Silico, and In Vitro Approaches
Source: Foods. 2023 Oct 1;12(19):3643. doi: 10.3390/foods12193643 (PMC10573055; doi:10.3390/foods12193643)
Supplement: Supplementary file 1 [file foods-12-03643-s001.zip › foods-2619679-supplementary.pdf]

foods

Supplementary data

Antioxidant and Anti-Inflammatory Mechanisms of Lipophilic Fractions from *Polyscias fruticosa* Leaves Based on Network Pharmacology, In Silico, and In Vitro Approaches

Razanamanana H. G. Rarison [1,†], Van-Long Truong [1,2,†], Byoung-Hoon Yoon [1], Ji-Won Park [1] and Woo-Sik Jeong [1,2,*]

[1] School of Food Science & Biotechnology, College of Agriculture and Life Sciences, Kyungpook National University, Daegu 41566, Republic of Korea;
[2] Food and Bio-industry Research Institute, School of Food Science & Biotechnology, College of Agriculture and Life Sciences, Kyungpook National University, Daegu 41566, Republic of Korea; rhanitranirina@gmail.com (R.H.G.R.); truonglongpro@gmail.com (V.-L.T.); byounghoon97@naver.com (B.-H.Y.); yozi6789@naver.com (J.-W.P.)
* Correspondence: wsjeong@knu.ac.kr; Tel.: +82-53-950-5775
† These authors contributed equally to this work.

Supplementary Table S1. The PDB ID and the box center coordinates for each protein.

Protein	PDB ID	Grid box center
KEAP1	6TYPA	center_x= -15.749371<br>center_y= 3.679657<br>center_z= 13.115057
NR1I2	6P2BA	center_x= 12.397710<br>center_y= 32.873097<br>center_z= 23.96642
PTGS2	5IKQ	center_x= 21.597621<br>center_y= 51.876966<br>center_z= 17.696379
PRKCD	1YRK	center_x= 25.614261<br>center_y= 43.053774<br>center_z= 21.103087
NFKB1	1SCV	center_x= 27.792003<br>center_y= 30.851276<br>center_z= 27.702665
TLR4	3FXI	center_x= 27.128832<br>center_y= -10.816630<br>center_z= 13.134016
NFE2L2	7X5E	center_x= -42.091138<br>center_y= -18.945142<br>center_z= 20.259611

Supplementary Table S2. Top 10 active compounds in PFL obtained from PFL compounds-target network.

Active compounds	Degree	Betweenness Centrality	Closeness Centrality
Falcarinol	62	0.01856039	0.43831169
(Z)-1,3-Phytadiene	61	0.02731567	0.4368932
Copaene	60	0.01315604	0.43548387
(3.beta.,5.alpha.)-Stigmasta-7,16-dien-3-ol	60	0.0148525	0.43548387
Ylangene	60	0.01315604	0.43548387
Alloisolongifolene alcohol	57	0.02326424	0.4313099
(+)-gamma-Cadinene	56	0.01389639	0.42993631
Neoclovene oxide	56	0.01298989	0.42993631
Stigmasterol	56	0.01056441	0.42993631
Actinidiolide, dihydro-	55	0.03256982	0.42857143

Supplementary Table S3. Top 15 target genes obtained from PFL compounds-target network.

Targets	Degree	Betweenness Centrality	Closeness Centrality
Nuclear factor NF-kappa-B p50 (NFKB1)	71	0.030183613	0.575692964
DNA-(apurinic or apyrimidinic site) lyase (APEX1)	70	0.02926807	0.573248408
Cyclooxygenase-1 (PTGS1)	60	0.018021206	0.526315789
Cytochrome P450 3A4 (CYP3A4)	60	0.019300353	0.532544379
Pregnane X receptor (NR1I2)	59	0.022330441	0.543259557
Acyl-CoA desaturase (SCD)	58	0.015428461	0.512333966
Nuclear factor erythroid 2-related factor 2 (NFE2L2)	55	0.020523533	0.532544379
Androgen Receptor (AR)	53	0.01393878	0.512333966
Casein kinase II alpha/beta (CSNK2B)	53	0.016134787	0.51625239
Protein-tyrosine phosphatase 1B (PTPN1)	52	0.015745039	0.51625239
Toll-like receptor 4 (TLR4)	49	0.010901582	0.491803279
Cyclooxygenase-2 (PTGS2)	48	0.013486764	0.504672897
Protein kinase C delta (PRKCD)	48	0.010764119	0.495412844
Aminopeptidase N (ANPEP)	48	0.012798254	0.497237569
Kelch-like ECH-associated protein 1 (KEAP1)	46	0.013118779	0.504672897

Supplementary Table S4. Molecular docking results of key active compounds in PFL and the hub genes

Protein	PBD ID	Compounds	Binding energy (kcal/mol)	Interactions	
				Alkyl/ Pi-Alkyl/Pi-Sigma/Unfavorable donor-donor/Carbon hydrogen bond/ Conventional hydrogen bond	Van der Waals
KEAP1	6TYP	Stigmasterol	-9.9	Ala366, Arg415, Val465, Ala556	Tyr334, Ser363, Gly364, Leu365, Gly367, Arg380, Asn414, Val418, Gly462, Val463, Gly464, Ala466, Gly509, Ala510, Gly511, Val512, Leu557, Ile559, Gly603, Val604, Gly605, Val606
		(3.beta.,5.alpha.)-Stigmasta-7,16-dien-3-ol	-9.4	Cys513, Ala556	Gly364, Leu365, Ala366, Gly367, Cys368, Arg415, Ile416, Gly417, Val418, Gly419, Val420, Gly462, Val463, Gly464, Val465, Ala466, Gly509, Ala510, Gly511, Val512, Val514, Leu557, Gly558, Ile559, Gly603, Val604, Gly605, Val606, Ala607
		Copaene	-6.7	Ala366	Leu365, Gly367, Ile416, Gly417, Val463, Gly464, Val465, Gly509, Ala510, Gly511, Val512, Ala556, Leu557, Gly558, Ile559, Gly603, Val604, Gly605, Val606
		(+)-gamma-Cadinene	-6.7	Tyr334, Ala556, Tyr572, Phe577	Ser555, Arg415, Arg380, Ser363, Ser603
		Ylangene	-6.9	Tyr334, Arg415, Ala556	Ser363, Gly364, Arg380, Tyr572, Phe577, Ser602, Gly603
		Alloisolongifolene alcohol	-7	Ala366, Gly462	Leu365, Gly367, Ile416, Gly417, Val418, Val463, Gly464, Val465, Gly509, Ala510, Gly511, Val512, Ala556, Leu557, Ile559, Val604, Gly605, Val606
		Neoclovene oxide	-7.3		Leu365, Ala366, Gly367, Ile416, Gly417, Val418, Val463, Gly464, Val465, Ala510, Gly511, Val512, Leu557, Gly558, Ile559, Val604, Val606
		Actinidiolide, dihydro-	-6.5	Gly367, Val606	Leu365, Ala366, Gly417, Val418, Val463, Gly464, Val465, Ala510, Gly511, Val512, Leu557, Gly558, Ile559, Val604, Gly605

		Falcarinol	-5.6	Ala366, Ile416, Val463, Val465, Val512	Gly364, Leu365, Gly367, Arg415, Gly417, Val418, Gly462, Gly464, Gly509, Ala510, Gly511, Ile559, Val604, Gly605, Val606
		(Z)-1,3-Phytadiene	-5.6	Tyr334, Ile461, Phe478, Tyr525, Ala556, Tyr572	Arg380, Arg415, Gly462, Arg483, Ser508, Gly509, Gln530, Ser555, Phe577, Ser602, Gly603
NR1I2	6P2B	Stigmasterol	-7.9	Lys170, Leu174, Leu215, Trp223	Phe172, Pro175, Val177, Leu213, Gly217, Glu218, Asn224, Tyr225, Arg303
		(3.beta.,5.alpha.)-Stigmasta-7,16-dien-3-ol	-6.9	Phe172, Trp223	Asn171, Leu174, Pro175, Val177, Leu215, Tyr225, Arg303
		Copaene	-8.7	Phe288, Trp299, Met323	Leu209, Val211, Met243, Gln285, Tyr306, Leu308, Leu324, His327
		(+)-gamma-Cadinene	-7.6	Leu209, Val211, Phe288, Trp299, Leu308, Leu324, His327	Met243, Gln285, Met323
		Ylangene	-8.6	Phe288, Trp299, Tyr306, Met323	Leu209, Val211, Met243, Gln285, Leu308, Leu324, His327
		Alloisolongifolene alcohol	-8.3	Val211, Trp299	Leu209, Met243, Gln285, Phe288, Tyr306, Leu308, Met323, Leu324, His327
		Neoclovene oxide	-8	Phe288, Trp299, Met323, His327	Leu209, Val211, Met243, Phe281, Gln285, Tyr306, His407
		Actinidiolide, dihydro-	-7.5	Val211, Trp299, His327	Leu209, Met243, Gln285, Phe288, Tyr306, Met323, Leu324
		Falcarinol	-6.4	Leu206, Leu209, Val211, Met243, Phe288, Trp299, Tyr306, Met323, Leu324, His327, His407	Leu240, Gln285, Leu308, Arg410
		(Z)-1,3-Phytadiene	-6.1	Leu206, Leu209, Val211, Leu240, Met243, Phe288, Trp299, Tyr306, Met323, His407	Gln285, Ile414, Phe420, Leu411, Leu324
PTGS2	5IKQ	Stigmasterol	-8.9	Leu294, Leu391, Tyr404, Ile408, Ala443, Val444	Ala202, Gln203, His207, Tyr385, His386, Trp387, His388, Leu390, Val447
		(3.beta.,5.alpha.)-Stigmasta-7,16-dien-3-ol	-8.8		Ala199, Ala202, Gln203, Thr206, His207, Leu294, Val295, Tyr385, Trp387, His388, Leu390, Leu391, Ile408, Val444, Val447
		Copaene	-8	Val349, Leu352, Tyr355, Val523, Ala527	Tyr348, Ser353, Phe381, Leu384, Tyr385, Trp387, Phe518, Met522, Gly526, Ser530

		(+)-gamma-Cadinene	-8.8	Val349, Leu352, Val523, Ala527, Leu531	Tyr348, Ser353, Phe381, Leu384, Tyr385, Trp387, Phe518, Met522, Gly526, Ser530
		Ylangene	-7	Tyr348, Val349, Leu352, Val523, Ala527	Phe281, Ser353, Leu384, Tyr385, Trp387, Phe518, Met522, Gly526, Ser530
		Alloisolongifolene alcohol	-6.3	Arg120, Glu524	Lys83, Pro84, Pro86, Val89, Tyr115, Ser119, His122, Leu123, Met471, Leu472
		Neoclovene oxide	-6.6	Val348, Leu352, Val523	Tyr348, Ser353, Tyr355, Trp387, Phe518, Met522, Gly526, Ala527, Ser530
		Actinidiolide, dihydro-	-6.7	Leu352, Val523, Ala527	Tyr348, Val349, Ser353, Phe381, Tyr385, Trp387, Phe518, Met522, Gly526, Ser530, Leu531
		Falcarinol	-7.1	Arg120, Val349, Leu352, Tyr385, Trp387, Phe518, Val523, Ala527	Val116, Tyr348, Ser350, Ser353, Tyr355, Leu359, Phe381, Leu384, Met522, Gly526, Leu531
		(Z)-1,3-Phytadiene	-7.1	Val116, Ile345, Tyr348, Val349, Leu352, Tyr355, Leu359, Val523, Ala527, Leu531	Met113, Arg120, Ser353, Phe381, Leu384, Tyr385, Trp387, Phe518, Met522, Gly526, Ser530
PRKCD	1YRK	Stigmasterol	-7.5	Phe27, Tyr52, Ala77	Gln25, Glu54, Trp55, Met74, Glu78
		(3.beta.,5.alpha.)-Stigmasta-7,16-dien-3-ol	-6.7	Phe27, Tyr52, Ala77	Gln25, Glu54, Trp55
		Copaene	-5.7	Phe27, Tyr52	Pro53, Glu53
		(+)-gamma-Cadinene	-6.1	Phe27	Tyr52, Pro53, Glu54
		Ylangene	-5.8	Phe27, Tyr52	Cys28, Pro53, Glu54
		Alloisolongifolene alcohol	-5.3	Gln25, Phe27, Trp55	Pro26, Tyr52, Glu54
		Neoclovene oxide	-5.2	Phe27	Tyr52, Pro53, Glu54, Trp55
		Actinidiolide, dihydro-	-4.8	Phe27	Gln25, Pro26, Tyr52, Pro53, Glu54, Trp55
		Falcarinol	-4.8	Pro26, Phe27, Tyr52	Gln25, Pro53, Glu54, Trp55
		(Z)-1,3-Phytadiene	-4.7	Phe27, Tyr52, Trp55, Ala77	Gln25, Pro26, Pro53, Glu54
NFKB1	1SCV	Stigmasterol	-7	Tyr60, Val61, Val145	Ala62, Glu63, Leu143, His144, Thr146, Lys149, Thr153, Ala156, Arg157
		(3.beta.,5.alpha.)-Stigmasta-7,16-dien-3-ol	-6.8	Phe56, Arg59, Lys80	Lys52, Gly55, Arg57, His67, Gly68, Gly69, Pro71, Ser74, Ser75, Glu76, Lys79, Ser81
		Copaene	-5.5	Val61, Leu143, Val145, Lys149	Ser113, Thr146, Thr153, Arg157
		(+)-gamma-Cadinene	-5.8	Val61, Leu143, Val145, Lys149	
		Ylangene	-5.4	Val61, Leu143, Val145, Lys149	Ser113, Thr146, Thr153, Arg157

		Alloisolongifolene alcohol	-5.4	Ser113, Leu143	Val61, Ala111, His112, His144, Val145, Thr146, Lys149, Thr153, Arg157
		Neoclovene oxide	-5.2	Lys275	Arg57, Ser243, Lys244, Ser249, Asn250, Asp274, Phe310
		Actinidiolide, dihydro-	-5.3	Val61, Ser113, Arg157	Ala111, His112, Leu143, Val145, Lys149, Thr153
		Falcarinol	-5.2	Phe56, Arg57, Arg59, Pro65, Val115, Leu143	Lys52, Gly55, Gly64, His67, Gly68, Pro71, Gly141, Ile142
		(Z)-1,3-Phytadiene	-5.1	Phe56, Arg59, Pro65, Val115, Lys117, Leu143	Tyr60, Val61, Gly64, His67, Gly68, Gly116, Asn139, Gly141, Ile142
TLR4	3FXI	Stigmasterol	-6.4	Ser360, His431, His458	Asn339, Asn361, Lys362, Arg382, Asn383, Gly384, Phe408, Asn409, Ser432, Asn433, His456, Thr457
		(3.beta.,5.alpha.)-Stigmasta-7,16-dien-3-ol	-5.8	Tyr295	
		Copaene	-5.5	Leu212	Pro214, Asn235, Asn236, Phe237, Asp238, Glu266, Gly267
		(+)-gamma-Cadinene	-5.3	Ala291, Val316	Arg234, Phe263, Arg264, Arg289, Tyr292, Ser317
		Ylangene	-5.3	Pro214	Leu212, Asn235, Phe237, Asp238, Glu266, Gly267
		Alloisolongifolene alcohol	-5.3	Asn339, Lys341	Arg264, Tyr292, Leu293, Asp294, Tyr296, Ser317, Val318, Thr319, Cys340, Lys362
		Neoclovene oxide	-5.6		Arg264, Tyr292, Leu293, Asp294, Tyr296, Ser317, Val318, Thr319, Lys341, Lys362
		Actinidiolide, dihydro-	-5.4	Tyr292, Ser317	Arg264, Leu293, Asp294, Tyr296, Val318, Thr319, Cys340, Lys341, Lys362
		Falcarinol	-4.2	Arg264, Tyr292, Tyr296	Asn268, Leu293, Asp294, Ser317, Val318, Thr319, Lys362
		(Z)-1,3-Phytadiene	-3.7	Leu212, Pro214	Asn235, Asn236, Phe237, Asp238, Glu266, Gly267
NFE2L2	7X5E	Stigmasterol	-6.6	Phe481, Arg499, Arg502, Arg503, Lys506	Asn482, Met485, Asn507, Ala510
		(3.beta.,5.alpha.)-Stigmasta-7,16-dien-3-ol	-6.1	Val478, Arg499, Arg503	Phe481, Arg502, Lys506
		Copaene	-6.1	Arg499, Arg502, Arg503	Lys506
		(+)-gamma-Cadinene	-6.2	Phe481, Arg499, Arg502, Lys506	Val478, Asn482, Arg503
		Ylangene	-6	Arg499, Arg502, Arg503	Lys506
		Alloisolongifolene alcohol	-5.5	Arg503	Arg499, Asp500, Arg502, Lys506

	Neoclovene oxide	-5.9	Arg503	Val478, Phe481, Arg499, Arg502, Lys506
	Actinidiolide, dihydro-	-5.4	Arg499, Arg502, Arg503	Phe481, Lys506
	Falcarinol	-4.7	Val478, Phe481, Arg499, Arg502, Arg503, Lys506	Asp500
	(Z)-1,3-Phytadiene	-4.2	Val478, Phe481, Arg499, Arg502, Arg503, Lys506	Asn482
